# Cost-benefit analysis of calcium and vitamin D supplements

**DOI:** 10.1007/s11657-019-0589-y

**Published:** 2019-04-30

**Authors:** Connie M. Weaver, Heike A. Bischoff–Ferrari, Christopher J. Shanahan

**Affiliations:** 1 Department of Nutrition Science, College of Health and Human Sciences, Purdue University, 700 W State Street, West Lafayette, IN 47907-2059 USA; 2 Department of Geriatric Medicine and Aging Research, University Hospital and University of Zurich, Rämistrasse 100, 8091 Zurich, Switzerland; 3 Centre on Aging and Mobility, University Hospital Zurich and Waid City Hospital, Zurich, Switzerland; 4 University Clinic for Acute Geriatric Care, Waid City Hospital, Zurich, Switzerland; 5 Frost & Sullivan, 7550 Interstate 10 Frontage Rd #400, San Antonio, TX 78229 USA

**Keywords:** Osteoporosis, Bone fracture, Calcium, Vitamin D, Costs and cost analysis, Cost savings

## Abstract

***Summary*:**

If all adults with osteoporosis in the European Union (EU) and United States (US) used calcium and vitamin D supplements, it could prevent more than 500,000 fractures/year in the EU and more than 300,000/year in the US and save approximately €5.7 billion and US $3.3 billion annually.

**Purpose:**

Evaluate the cost-effectiveness of calcium/vitamin D supplementation for preventing osteoporotic fractures.

**Methods:**

A cost-benefit analysis tool was used to estimate the net cost savings from reduced fracture-related hospital expenses if adults with osteoporosis in the EU and US used calcium/vitamin D supplements. A 14% relative risk reduction of fracture with calcium/vitamin D supplementation from a recent systematic review and meta-analysis of randomized, controlled trials was used as the basis for the benefit estimate. Other model inputs were informed by epidemiologic, clinical, and cost data (2016–2017) obtained via the medical literature or public databases. Analyses estimated the total number of avoided fractures and associated cost savings with supplement use. Net cost benefit was calculated by subtracting the supplements’ market costs from those savings.

**Results:**

The > 30 million persons in the EU and nearly 11 million in US with osteoporosis experience about 3.9 million and 2.3 million fractures/year and have annual hospital costs exceeding €50 billion and $28 billion. If all persons with osteoporosis used calcium and vitamin D supplements, there would be an estimated 544,687 fewer fractures/year in the EU and 323,566 fewer in the US, saving over €6.9 billion and $3.9 billion; the net cost benefit would be €5,710,277,330 and $3,312,236,252, respectively.

**Conclusions:**

Calcium and vitamin D supplements are highly cost-effective, and expanded use could considerably reduce fractures and related costs. Although these analyses included individuals aged ≥ 50 years, the observed effects are likely driven by benefits observed in those aged ≥ 65 years.

**Electronic supplementary material:**

The online version of this article (10.1007/s11657-019-0589-y) contains supplementary material, which is available to authorized users.

## Introduction

According to the International Osteoporosis Foundation, osteoporosis is a worldwide epidemic [1]. Osteoporotic fractures are often the first sign of the disease [2], and frequently occur after a low-impact injury or fall, but vertebral fractures can also occur during routine activities in the absence of a fall or injury [2, 3]. Fractures result in pain, deformity, height loss, impaired quality of life, restricted mobility, loss of independence, and, especially in the case of hip fractures, increased mortality [2, 3].

Recommended strategies for fracture prevention include, among other things, adequate consumption of calcium and vitamin D [2, 3]. Mineralization of bone requires calcium, and dietary calcium absorption in the gut requires the presence of vitamin D [4]. Vitamin D also supports bone growth and remodeling by osteoblasts and osteoclasts [4]. For persons aged 51 years and older, United States (US) dietary guidelines recommend a daily calcium intake of 1200 mg for women and 1000 mg for men and a daily vitamin D intake of 600 mg for both genders [5]. However, most North American osteoporosis guidelines recommend a vitamin D intake of at least 800 to 1000 IU/day (maximum 4000 IU/day) for adults aged 50 years and older [2, 3, 6, 7]. European guidelines vary by country, but a daily intake of at least 1000 mg of calcium, which includes dietary sources of calcium, and 400 to 800 IU of vitamin D is generally recommended [8, 9].

Unfortunately, calcium and especially vitamin D are both underconsumed dietary nutrients [5], and inadequacies in these nutrients are a key preventable risk factor for osteoporosis [10]. Among persons who rely on food alone for their vitamin and mineral intake, about 38% consume inadequate levels of calcium and about 93% consume inadequate levels of vitamin D [11]. Vitamin D is also produced in the skin in response to sunlight, but sunshine exposure may be limited by climate or sunscreen use [5]. About 58% of persons in Europe have serum 25-hydroxyvitamin D levels < 30 ng/mL [12].

It remains controversial as to whether increasing dietary calcium intake results in clinically meaningful fracture reduction [13–15]. Also, calcium supplementation alone does not reduce fracture risk compared with placebo [16]. However, calcium and vitamin D supplementation is associated with a 14% reduction in overall risk of fractures and a 39% reduction in risk of hip fractures in generally healthy adults, according to a recent meta-analysis [4]. Most professional guidelines from North America and the European Union (EU) support the use of supplements to maintain adequate calcium and vitamin D intake [2, 3, 6, 7, 9].

Expanded use of combined calcium and vitamin D supplements has the potential to lower the risk of fracture. The financial impact of such prevention efforts is unclear, however, since the supplements have an associated cost and would need to be used by a large number of people to prevent one fracture. This analysis evaluated the net cost savings that could be derived from reduced hospital expenses for bone fracture if all adults aged 50 and older with osteoporosis in the EU and the US used calcium and vitamin D supplements, assuming a 14% reduction of total fracture risk [4]. We hypothesized that, despite the costs of the supplements, savings would be derived from a reduction in fractures.

## Methods

### Target populations

This analysis calculated fracture risk reduction and cost savings from calcium and vitamin D supplements in target populations consisting of adults aged 50 and older with osteoporosis in the EU and US. In addition, analyses were performed to evaluate risk reduction and cost savings in subgroups of those populations by age (50–59 years, 60–69 years, 70–79 years, and > 80 years) and gender. All analyses were performed by Frost & Sullivan (San Antonio, TX, USA).

### Identification of fracture risk and related costs

We derived overall EU and US population data, as well as population data for individual countries and states, from Eurostat [17], the US Census Bureau [18], and the Kaiser Family Foundation [19]; data were adjusted to align with the 10-year age bands of the target populations and were also sorted by gender. We modeled the current prevalence of osteoporosis by applying osteoporosis prevalence data from Hernlund et al. [8] for the EU and Wright et al. [20] for the US to current total population figures. Gaps in information, including missing EU countries, were approximated using similar findings from the nearest and most similar country with complete information.

We modeled the number of osteoporosis-attributed fractures using reported annual fracture incidence rates derived from Kanis et al. [21] and Johnell et al. [22] for the EU and Burge et al. [23] for the US. Our estimates are conservative in nature because the data from Kanis et al. [21] are for hip fractures only. As US state-level fracture rates were not available, we first applied the osteoporosis prevalence rates by state from Wright et al. [20] to distribute expected osteoporosis-attributed bone fractures. Relative incidence rates of osteoporosis-attributed fractures for each EU country and US state were then applied to current population data to obtain estimated current osteoporosis-attributed bone fracture rates. Future survey research is required to verify the latest osteoporosis fracture rates. The incidence of fracture (*F*) in each target population was then calculated as the number of fractures per year/number of people with osteoporosis.

We modeled the estimated per-person and total annual costs of an osteoporosis-attributed bone fracture using reported costs from Hernlund et al. [8] for the EU and Weycker et al. [24] and Burge et al. [23] for the US. Since the study by Kanis et al. reported costs for base year 2010, we scaled these figures upward to account for inflation, assuming a 2% annual growth in per-fracture cost from 2010 to 2016. Purchasing power parity (PPP) per EU country and US state was used to further control and adjust for relative healthcare costs between countries and states. We relied on the data from Burge et al. [23] to segment costs by gender, age, and US state and adjusted for target population growth. We calculated the cost equivalent for each of the estimated 2.3 million total fractures in 2016 by multiplying the average cost of fracture (US $12,197) times the PPP of each US state and a cumulative inflation rate of 43.7% between 2005 and 2016 (consistent with a conservative estimate of about 3.6% annual growth of the US healthcare spending [25]). Future survey research is required to verify the estimated annual cost of an osteoporosis-attributed bone fracture.

### Determination of expected impact of calcium/vitamin D supplements on fracture risk

A search of the medical literature identified a recent, comprehensive meta-analysis of 7 randomized, controlled trials [26–32] of calcium (500–1200 mg/day) plus vitamin D (400–800 IU/day) supplementation for fracture prevention in generally healthy community-dwelling and institutionalized adults conducted by the National Osteoporosis Foundation (Table 1) [4]. The final analysis, published in a correction, reported a summary relative risk estimate of 0.86 (95% CI, 0.75–0.98), indicating that supplementation would reduce the overall population risk of osteoporotic fracture by 14% [4]. Thus, the current analysis utilized a relative risk reduction (RRR) for total fracture of 0.14 with calcium and vitamin D supplementation.

**Table 1:** Summary of randomized, controlled trials included in meta-analysis for calcium plus vitamin D supplementation versus control and fracture risk reduction. Adapted by permission from Springer Customer Service Centre GmbH: Springer Nature, *Osteoporosis International*. Erratum and additional analyses re: Calcium plus vitamin D supplementation and the risk of fractures: an updated meta-analysis from the National Osteoporosis Foundation. Weaver CM, Dawson-Hughes B, Lappe JM, Wallace TC, 2016 [4]

Study	Region	Population	*N*	Gender, *n* (%)		Age, year (range) Mean (SD) Tx Control	Calcium dose, mg/day	Vitamin D dose, IU/day	Number of fractures, *n*/*N*		Calculated RR (95% CI) [4]
				Women	Men				Supplements	Control	
Chapuy 1992 [26]	France	Institutionalized	3270	3270 (100)	0	(69–106) 84 (6) 84 (6)	1200	800	66/877	97/888	0.69 (0.51–0.93)
Dawson-Hughes 1997 [27]	USA	Community dwelling	445	246 (55.3)	199 (44.7)	(≥ 65)^a^ Women: 71 (4) 72 (5) Men: 70 (4) 71 (5)	500	700	11/170	26/148	0.37 (0.19–0.72)
Porthouse 2005 [28]	England	Community dwelling, unequally allocated group (2:1)	3454	3454 (100)	0	(≥ 70)^a^ 77 (5) 77 (5)	1000	800	34/714	69/1391	0.96 (0.64–1.43)
		Community dwelling, equally allocated group (1:1)							24/607	22/602	1.08 (0.61–1.91)
Prentice 2013^b^ [29]	USA	Community dwelling	15,331	15,331 (100)	0	(50–79) NR NR	1000	400	405/7530^c^	458/7801^c^	0.92 (0.80–1.04)
Salovaara 2010 [30]	Finland	Community dwelling	3195	3195 (100)	0	(65–71) 67 (2) 67 (2)	1000	800	78/1586	94/1609	0.84 (0.63–1.13)
Grant 2005^d^ [31]	UK	Community dwelling with history of fracture	2638^e^	2232 (84.6)	406 (15.4)	(≥ 70)^a^ 78 (6) 77 (6)	1000	800	179/1306	192/1332	0.95 (0.79–1.15)
Harwood 2004 [32]	UK	Community dwelling with history of hip fracture	76^f^	76	0	(67–92) 83 81^g^	1000	800	3/29	5/35	0.72 (0.19–2.78)
Total (random model) [4]									800/12,819	963/13,806	0.86 (0.75–0.98)

^a^Range not reported; number in parentheses represents age-related inclusion criteria

^b^Data analyzing the WHI for subgroup with adherence to assigned pills and no personal use of supplements, from Table 6 in Prentice et al. 2013 [29]

^c^Data provided from WHI investigators

^d^Results from data on fracture confirmed by radiography

^e^A total of 5292 were randomized to four groups: 1306 to calcium and vitamin D, 1343 to vitamin D alone, 1311 to calcium alone, and 1332 to placebo; only the calcium + vitamin D and placebo groups are included here

^f^A total of 150 women were randomized to four groups: 38 to injected vitamin D, 36 to injected vitamin D with oral calcium; 39 to oral vitamin D and calcium, and 37 to control; only the oral vitamin D and calcium and control groups are included here

^g^SDs not reported

*CI*, confidence interval; *NR*, not reported; *RR*, relative risk; *SD*, standard deviation; *Tx*, treatment; *WHI*, Women’s Health Initiative

The RRR (0.14) was used to calculate the absolute risk reduction realizable for each target population by multiplying it by the target population’s incidence of an osteoporosis-attributed bone fracture (*F*). Number needed to treat (NNT)—i.e., the total number of people who would have to be treated with the supplements to achieve one avoided fracture event—was calculated as 1/(*F* – [*F* · *RRR*]).The number of avoided fractures (N*) was calculated as the number of people in the target population with osteoporosis multiplied by the absolute risk reduction or the inverse of NNT.

### Calculation of net realizable healthcare cost savings

A previously published cost-benefit analysis tool developed by researchers at Frost & Sullivan was used for this analysis [33]. The following equation was used to calculate total potential savings (*S*) from reduced hospital services following osteoporosis-attributed fracture that would be realizable if the entire target population used a calcium and vitamin D supplement: *S* = *h* · *N*∗ where *h* is the expected per-person cost of an osteoporosis-attributed bone fracture for a member of the target population (*Pop*) derived as described above.

It is also necessary to take into account the cost of the calcium and vitamin D supplement. Therefore, the net cost benefits (*B*) that could be realized from avoided osteoporosis-attributed bone fractures were calculated as *B* = *S* − *C* = *S* − (*Pop* · *d* ), where cost savings *S* is calculated as described above and the total population cost (*C*) of calcium and vitamin D supplementation is represented by *C = Pop* · *d* where *Pop* is the total number of at-risk people in the target population and *d* is the expected annual per-person cost for a 1000-mg/day calcium and 600-IU/day vitamin D supplement regimen. In order to have a reasonable estimate of savings, we based supplement costs on calcium and vitamin D doses at the relatively higher end of the usual dose ranges studied in the meta-analysis on which the RRR was based; these regimen levels represent mid to high doses of supplements typically available to consumers from the marketplace. A vitamin D dose of 600 IU (15 μg) per day has also been established by the European Food Safety Authority as adequate intake for adults to achieve a target serum 25-hydroxyvitamin D concentration of 50 nmol/L [34]. Supplement cost (*d*), which represents the average cost across a wide range of available supplements, was determined based on market analysis performed by Frost & Sullivan (unpublished data, 2017).

Note that the entire target population must take the given regimen in order for the total number of avoided events to be realized. Thus, *B* represents the net monetary benefits that use of calcium and vitamin D supplements can yield through hospital cost reduction with 100% utilization of supplements by the target population.

## Results

### Osteoporosis, fractures, and associated hospital costs

In the EU, an estimated 30,485,309 persons aged 50 years and older have osteoporosis, representing nearly 15% of persons in that age range (Table 2). In the US, an estimated 10,887,910 persons aged 50 years and older have osteoporosis, representing about 10% of the US population of that age (Table 2). Women account for about 80% of those with osteoporosis in both the EU and the US. Osteoporosis rates increase with age in both regions and are about 7%, 12%, and 24% among persons in their 50s, 60s, and 70s in the EU and 5%, 8%, and 16% in those age groups in the US, respectively. In both regions, a little more than a quarter of those aged over 80 years have osteoporosis.

**Table 2:** Osteoporosis-attributed fractures and related hospital expenditures in adults aged ≥ 50 years in the European Union and United States, by age and gender, 2016–2017

	Gender		Age, year				All women and men > 50 years of age
	Women	Men	50–59	60–69	70–79	≥ 80	
European Union							
Overall population, *n*	110,412,069	94,341,393	72,894,061	61,182,643	42,352,016	28,324,742	**204,753,462**
Number (%) of people with osteoporosis	24,289,066 (22.0)	6,196,243 (6.6)	5,400,985 (7.4)	7,089,079 (11.6)	10,096,028 (23.8)	7,899,217 (27.9)	**30,485,309** (**14.9**)
Number of osteoporosis-attributable fractures/year	2,582,774	1,341,884	695,781	908,098	1,313,006	1,007,773	**3,924,658**
Annual percentage of osteoporotic population who experience a fracture	10.6	21.7	12.9	12.8	13.0	12.8	**12.9**
Per-person hospital-related cost of fracture, €	–	–	–	–	–	–	**12,772**
Total annual hospital costs of osteoporosis-attributed fractures, €^a^	33,305,134,948	16,818,991,244	8,886,229,029	11,597,857,669	16,769,170,031	12,870,869,463	**50,124,126,192**
United States							
Overall population, *n*	56,943,826	49,204,489	44,980,229	31,374,453	17,789,749	12,005,722	**106,148,314**
Number (%) of people with osteoporosis	8,772,758 (15.4)	2,115,151 (4.3)	2,296,013 (5.1)	2,511,839 (8.0)	2,917,720 (16.4)	3,146,201 (26.2)	**10,887,910** (**10.3**)
Number of osteoporosis-attributable fractures/year	1,655,814	675,591	492,660	517,406	634,765	686,574	**2,331,405**
Annual percentage of osteoporotic population who experience a fracture	18.9	31.9	21.5	20.6	21.8	21.8	**21.4**
Per-person hospital-related cost of fracture, US $	–	–	–	–	–	–	**12,197**
Total annual hospital costs of osteoporosis-attributed fractures, US $^a^	20,196,239,923	8,240,299,495	6,009,057,865	6,310,891,878	7,742,336,469	8,374,253,206	**28,436,539,418**

The significance of the bold entries was to separate the overall totals from the individual age groups listed in Table 2, as well as the call out totals of each row

^a^Per-person hospital costs of fracture multiplied by the number of osteoporosis-attributable fractures per year

The estimated annual fracture incidence among persons with osteoporosis is 12.9% in the EU and 21.4% in the US, which translates to about 3.9 million fractures per year in Europe and 2.3 million per year in the US (Table 2), respectively. Although men have a lower risk of osteoporosis, those with osteoporosis have a higher risk of fracture compared with women with osteoporosis: 21.7%/year vs 10.6%/year in the EU and 31.9% vs 18.9% in the US. Among persons with osteoporosis, fracture rates appear to be similar across age groups, at about 12–13% in the EU and 21–22% in the US.

In the EU, osteoporosis-related fractures result in hospital costs of about €12,772 per person and over €50 billion annually (Table 2), with women accounting for 66% of cases. Hospital costs for osteoporosis-related fractures in the US are about $12,197 per person and over $28 billion annually (Table 2), with women accounting for about 71% of costs. Those aged ≥ 80 years account for about 26% of the total osteoporotic fracture–related hospital costs in the EU and 29% in the US.

### Fracture reduction with calcium and vitamin D supplementation

**Fig. 1:**
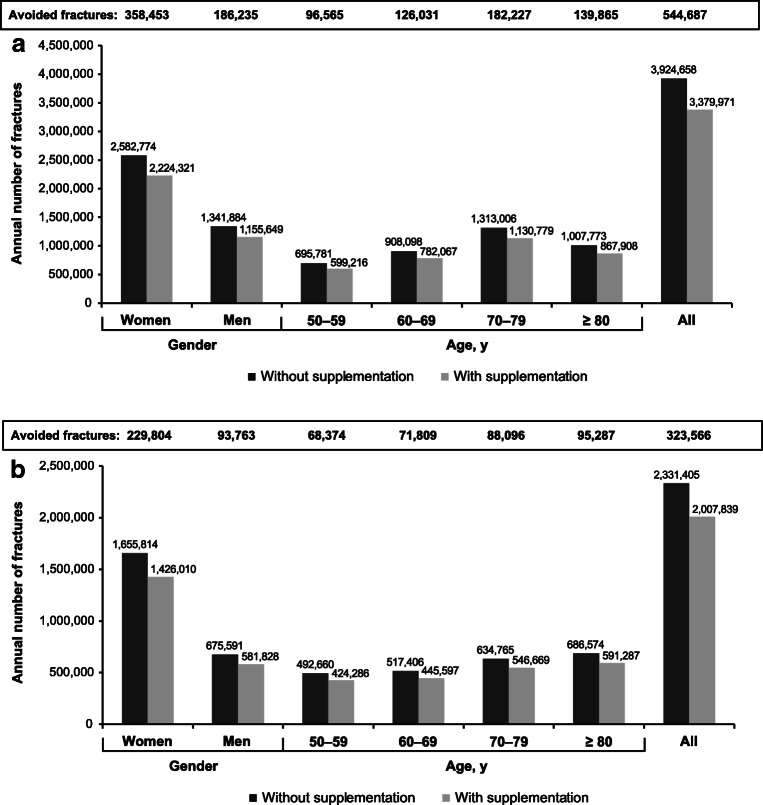
Estimated number of fractures avoided annually, by gender and age, with 100% use of calcium and vitamin D supplementation by adults aged ≥ 50 years in **a** the European Union and **b** the United States

Fifty-six people in Europe, or 34 people in the US, would need to be treated with calcium and vitamin D supplements to prevent one fracture in each of those target populations. Absolute risk reduction would be 1.8% in Europe and 3.0% in the US. If everyone aged 50 years and older took calcium and vitamin D supplements, an estimated 544,687 fractures would be prevented annually in the EU (Fig. 1a) and 323,566 would be prevented annually in the US (Fig. 1b). The number of avoided fractures would be higher in women than in men and would be highest among those in their 70s in the EU and in those ≥ 80 years old in the US compared with the other age groups (Fig. 1a and b).

### Cost savings with calcium and vitamin D supplementation

**Fig. 2:**
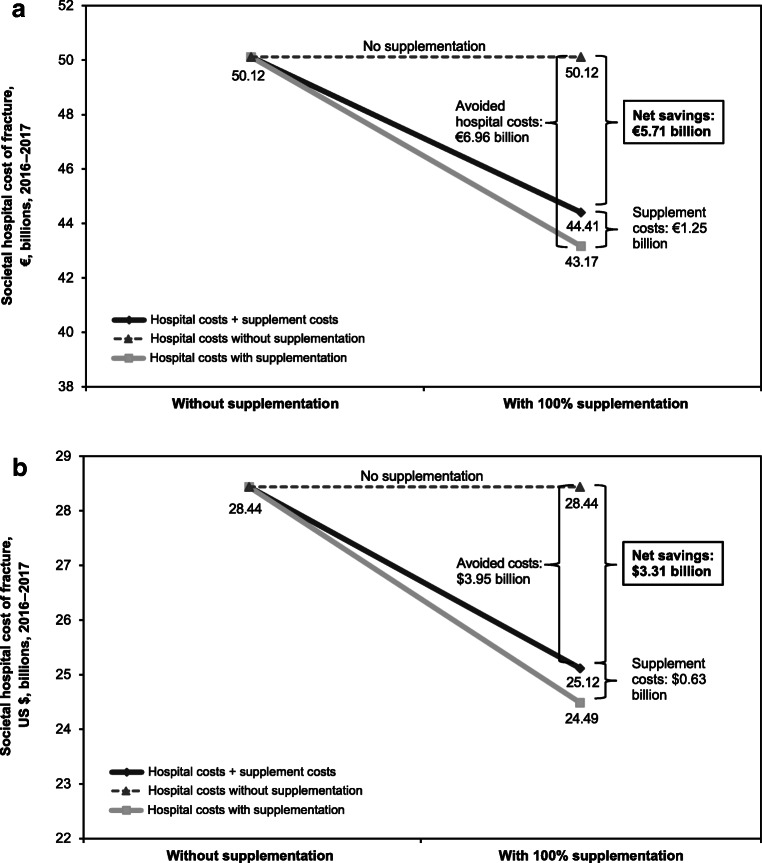
Cost analysis: net healthcare cost savings in terms of reduced hospital costs for osteoporosis-attributable fracture if all persons aged ≥ 50 years used calcium and vitamin D supplements in **a** the European Union and **b** the United States, 2016–2017

The reduction in fractures with calcium and vitamin D supplementation would save nearly €7 billion in the EU and nearly $4 billion in the US annually (Table 3). Factoring in the cost of the supplements, this would result in a net cost benefit of approximately €5.7 billion (Fig. 2a) and $3.3 billion (Fig. 2b) per year, respectively. The EU would save €5.58 in hospital costs for each €1 spent on calcium and vitamin D supplements, and the US would save $6.22 per $1 spent (Table 3). Breakdowns of the net cost/savings ratio by EU country and US state are provided in the Electronic Supplemental Materials (Online Resource 1), which also contain a breakdown of the overall cost analysis by EU country (Online Resource 2) and US state (Online Resource 3). Additionally, data describing osteoporosis-attributed fractures and related hospital expenditures in adults aged ≥ 50 years in the EU by age and gender are included in Online Resource 4; these data were not available for the US.

**Table 3:** Calcium and vitamin D supplementation cost benefit analysis, 2016–2017

	European Union, €	United States, US $
Cost savings (avoided hospital costs for osteoporosis-attributable fractures)		
Total annual savings (S)	6,956,520,691	3,946,589,993
Per-person annual savings	228.19	362.47
Costs of calcium and vitamin D supplements		
Total annual cost (C) per target population	1,246,243,361	634,353,741
Daily per-person cost	0.11	0.16
Annual per-person cost	40.88	58.44
Net cost benefits		
Annual net cost benefit (S – C)	5,710,277,330	3,312,236,252
Net benefit/cost ratio (S/C)	5.58	6.22

**Fig. 3:**
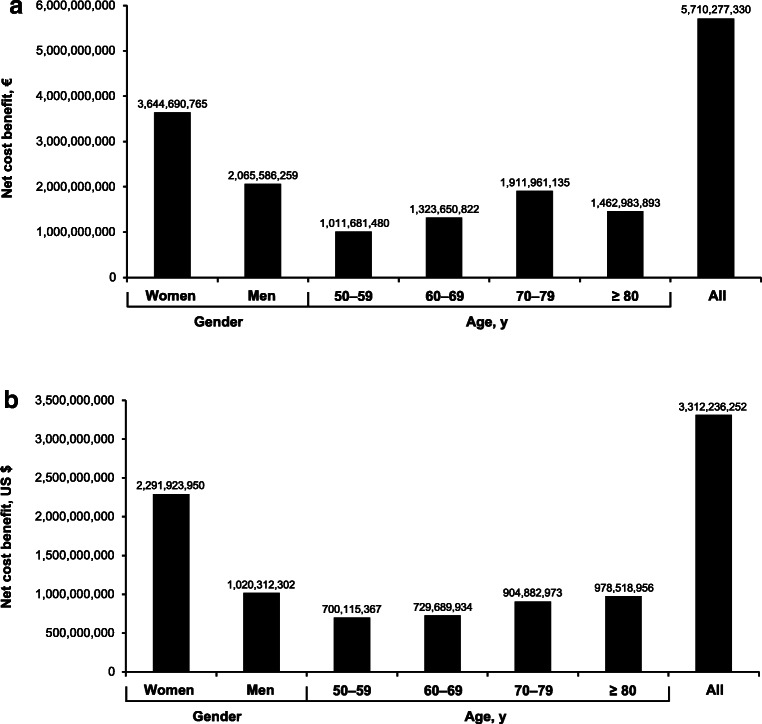
Net cost benefits, by gender and age, with 100% use of calcium and vitamin D supplementation by adults aged ≥ 50 years in **a** the European Union and **b** the United States

Fracture reductions in women would account for over €3.6 billion (~ 64%) of the cost savings in the EU (Fig. 3a) and nearly $2.3 billion (~ 69%) of the cost savings in the US (Fig. 3b). Cost savings associated with calcium and vitamin D supplementation increase with age in both populations up to age 80 and then begin to plateau or decline due to diminishing population levels for those in the age ≥ 80 cohort (Fig. 3a and b).

## Discussion

Dietary supplements are a simple, noninvasive measure with potential preventive health benefits. The current analysis supports our hypothesis and suggests that expanding the use of these supplements to 100% of adults aged ≥ 50 years could reduce the annual number of fractures by 544,687 in the EU and 323,566 in the US, resulting in a net cost benefit of over €5.71 billion and $3.31 billion, respectively, in avoided fracture-related hospital costs. Thus, it can be concluded that calcium and vitamin D supplements are highly cost-effective. However, it is important to note that the benefits observed in the overall population may in part be driven by greater reductions in the older populations, in particular in those aged ≥ 65 years, who comprise the majority of the population used to calculate the risk reduction, have higher risks for osteoporosis-related fractures, and may be more detrimentally affected by vitamin D deficiencies. As noted above, the benefits increased with age in these analyses. These findings may assist persons with osteoporosis, their healthcare providers, government and private insurance companies, and employers in making decisions or recommendations that could help minimize current and future fracture risks and related costs.

Fracture reduction and cost savings were predicted for both genders and across all age groups 50 years and older; however, increased use of calcium and vitamin D supplementation by women, particularly older women, has the potential to produce the greatest benefits with regard to fracture reduction and cost savings. One caveat is that our analysis applied a 14% reduction in fracture risk with supplementation across all age groups and both genders. The 14% total fracture reduction was derived from the recent meta-analysis by Weaver and colleagues [4]; most of the individuals enrolled in the studies included in that meta-analysis were 65 years of age and older, and only 1 study contributed data from women aged 50 years and older (Table 1) [29]. Therefore, future cost-benefit analyses in this area may benefit from additional analyses that control for age and determine the benefits for the different age groups evaluated. Also, the studies included in the Weaver et al. meta-analysis included fewer men, and the authors did not attempt to determine whether RRR varies by age or gender. Although men have a lower baseline risk of fracture [35], there is evidence in other studies and meta-analyses that they derive a benefit from calcium and vitamin D supplementation similar to women [31, 36]. Further studies are needed to confirm whether persons in their 50s derive the same benefits from calcium and vitamin D supplementation in terms of fracture reduction as older persons; it is possible that by applying the same RRR across all age groups, we have overestimated benefits in this younger age group and also underestimated benefits in the oldest groups. In addition, it should be noted that the original meta-analysis evaluated fracture reduction with calcium and vitamin D supplements in generally healthy adults, whereas our cost analysis is limited to persons with osteoporosis, who represent a high-risk subgroup.

A previous cost analysis predicted that costs of osteoporosis-related fracture in the US would grow from $16.9 billion in 2005 to $25.3 billion in 2025 [23]. Similarly, another previous cost analysis estimated that overall costs of osteoporotic fracture in Europe would increase from approximately €37.4 billion in 2010 to nearly €46.8 billion by 2025 [8]. Using their initial costs as a base and adjusting for price inflation and PPP between US states and EU countries, we estimated that hospital costs related to osteoporotic fractures have already exceeded $28.4 billion in 2016–2017 in the US and €50.1 billion in Europe. Future survey research is required to verify the estimated annual cost of an osteoporosis-attributed bone fracture. Another analysis predicted that adequate dairy intake containing 1000 to 1500 mg/day calcium could provide a 20% reduction in fracture risk and cost savings totaling $3.5 billion per year [37]; however, a subsequent meta-analysis found no overall association between dairy consumption and hip fracture risk [15].

It should be noted that the 14% RRR may be a conservative estimate of fracture reduction with calcium and vitamin D supplements. An earlier meta-analysis found vitamin D supplements at doses of 482–770 IU/day with or without calcium supplements were associated with a 20% reduction in all nonvertebral fractures and an 18% reduction in hip fracture [36]. In addition, the meta-analysis we used to derive the RRR included studies of supplements containing 500–1200 mg/day of calcium and 400–800 IU/day of vitamin D [4]. On the other hand, we may have overestimated a benefit of calcium in combination with vitamin D given the most recent meta-analysis by Zhao and colleagues [38], in which the authors found only a 10% nonsignificant reduction in total fracture risk with these supplements (risk ratio = 0.90; 95% CI, 0.78–1.04). However, 1 of the 8 trials included in that meta-analysis had a follow-up of less than 12 months, which is too short for fracture benefits to be expected; half of the studies had no control intervention; and the focus was on individuals aged ≥ 50 years, which may have lessened the impact that may have been observed in an older population. These limitations may have prevented the authors from documenting a benefit. Further, the most recent meta-analyses by Bolland and colleagues, which concluded there was no benefit of vitamin D on fracture risk, excluded all trials of vitamin D plus calcium [39, 40].

Our analysis was based on costs of supplements containing 1000 mg/day calcium and 600 IU/day vitamin D. Additional analyses to determine the exact impact of calcium and vitamin D supplement dose on the relative risk of fracture and related costs should be considered for future investigation, especially since benefits of vitamin D supplementation have been found to be dose related [36].

There are a number of challenges inherent to studies of nutritional interventions. For example, control groups are not truly untreated since they still have some level of intake from diet, as well as production of vitamin D from sun exposure, and possible intake of other dietary supplements containing these nutrients. The underlying meta-analysis by Weaver and colleagues [4] and the current cost analysis were not adjusted for baseline dietary or supplemental calcium and vitamin D intake. Data from the National Health and Nutrition Examination Survey (NHANES) show that usual intake among US adults age 19 years and older is 1061 ± 15 mg calcium from food alone versus 1277 ± 1 mg from food and supplements among supplement users; usual vitamin D intake is 5.17 ± 0.16 μg from food alone versus 17.1 ± 0.3 μg from food and supplements [11]. It can be seen from these NHANES data that supplements often help bring calcium and vitamin D intake closer to recommended levels.

Thereby, we note that the assumption of the current model that each target population shifts from zero to 100% usage of calcium and vitamin D supplements may overestimate the expected cost-benefit, as we do not take into account that each target population already includes some proportion of persons who are regular users of calcium and vitamin D supplements. Such individuals have therefore already realized the potential risk- and cost-reducing benefits of these supplements. A 2017 survey of 2001 US adults (age 18 years and older) by the Council for Responsible Nutrition found that 20% were using calcium supplements and 28% were using vitamin D supplements [41]. Thus, as a rough estimate, assuming that the 20% of calcium supplement users were all also vitamin D supplement users and that a similar proportion of the subgroup of persons aged 50 years and older were regular users, then a conservative estimate would be that at least 80% of persons in this age group have not yet realized the benefits of regular use. This still amounts to an unrealized potential reduction of 258,852 fractures and $2,649,789,001 net cost benefit in the US. Future investigations should include sensitivity analyses using different estimates of risk reduction and taking into account the impact of adherence and personal supplement use on both fracture risk reduction and supplement cost.

This cost model is based on 100% adherence, which represents an ideal scenario not typical of real-world use. Nonetheless, this model provides information as to maximum potential benefit from calcium and vitamin D supplements in the target populations, with the understanding that actual net benefits would be lower with lower rates of supplement uptake or less than 100% adherence among supplement users.

Currently, there is some controversy as to whether meta-analyses of calcium and vitamin D supplements should include all subjects (intent-to-treat population) or only those who were adherent during clinical trials [4, 29, 42]. Data from the Women’s Health Initiative (WHI) studies have been analyzed using multiple approaches: an intent-to-treat approach that evaluated the total population irrespective of adherence, a subgroup analysis that included only those subjects who were not taking personal supplements at baseline, and a per-protocol analysis that included only subjects who were not using supplements at baseline and who then adhered to the assigned supplement [29]. Not surprisingly, since treatments can only work if they are taken, the third approach provided the most compelling support for fracture reduction with calcium and vitamin D supplementation [29]. The meta-analysis used to determine RRR for the current cost analysis included the latter of these three groups from the WHI, and sensitivity analyses conducted using the other WHI analysis populations produced fairly similar results [4]. Data on fracture reduction in the subgroup of adherent participants were lacking in other studies included in the meta-analysis; however, it should be noted that the 14% reduction in fracture was seen even with less than 100% adherence in all of the trials [4]. In fact, reported adherence rates were as low as 55% to 63% in some of the included trials [28, 31]. Thus, the RRR used in this analysis is likely a conservative estimate of the benefit of supplements, given that those in the placebo group still have some baseline exposure to calcium and vitamin D and those in the treated group were not fully adherent.

The RRR for fracture in this analysis was derived from a meta-analysis in healthy community-dwelling and institutionalized adults and applied to a subgroup of such persons with osteoporosis; actual RRR may be higher or lower in this subgroup. In addition, since a majority of fragility fractures occur in persons with bone mineral density in the osteopenic range [43], additional cost analyses are needed to identify potential cost savings if calcium and vitamin D supplementation was expanded to include those with osteopenia, especially at elevated risk based on Fracture Risk Assessment Tool thresholds (e.g., ≥ 20% 10-year probability for major osteoporotic fracture or ≥ 3% 10-year probability of hip fracture in the US) [44].

The current analysis measures total benefits and does not necessarily predict fracture risk and savings for individual persons whose potential benefits vary with their specific risk of osteoporosis-related fracture. Individual fracture risk is dependent on a variety of factors such as age, race, gender, body mass index, bone mineral density, previous fracture history, health conditions (e.g., rheumatoid arthritis, hyperthyroidism, chronic liver disease), concurrent medications (e.g., glucocorticoids), and alcohol and tobacco use [44]. Evaluating the effects of calcium and vitamin D supplements on fracture risk would benefit from also evaluating their effects on bone quality. In addition, cost benefits might differ if looked at from either a patient or a payer perspective instead of a societal perspective. In real-world settings, actual costs are borne by a combination of insurers and patients. While insurers probably cover a majority of the hospital-based costs associated with fracture, patients likely bear the out-of-pocket costs of over-the-counter supplements, although in Europe, individual countries (e.g., Switzerland and Germany) reimburse the costs via health insurance claims for patients with established osteoporosis. Theoretically, if the out-of-pocket costs of the supplements were shifted to insurers, the removal of a cost barrier for patients could potentially lead to increased supplement use and associated decreases in fractures that would still result in cost savings for insurers based on the current analysis.

Only direct costs of hospitalization related to fracture were included in this cost-benefit analysis. Thus, the analysis does not take into account impact on other medical expenses (e.g., long-term care) or indirect costs (e.g., absenteeism and loss of productivity) related to fracture, nor does it take into account the impact to society of potential lost tax revenue. Some of those costs can be extensive (e.g., the cost of long-term care for disability from osteoporosis was €10.7 billion in Europe in 2010 [1]). Therefore, additional cost benefits beyond those measured in this analysis are potentially realized if fractures are reduced through expanded supplementation use.

As global populations continue to age, a considerable increase in osteoporosis prevalence is anticipated [1]. In fact, the number of individuals over the age of 50 years worldwide who are at high risk of osteoporotic fracture is expected to rise by about 30% in Europe and more than 50% in North America from 2010 to 2040 [45]. The current analysis suggests that expanding combined use of calcium and vitamin supplements among adults in this age group has the potential to reduce the risk of fractures and substantially reduce hospital costs for osteoporosis-attributable fractures. Potential cost savings based on 2016–2017 data amount to over €5.71 billion and nearly $3.31 billion per year in Europe and the US, respectively.

## Electronic supplementary material

ESM 1 (PDF 93 kb)

ESM 2 (PDF 17 kb)

ESM 3 (PDF 19 kb)

ESM 4 (PDF 15 kb)
