# Supplementary material for: Cost-benefit analysis of calcium and vitamin D supplements
Source: Arch Osteoporos. 2019 Apr 30;14(1):50. doi: 10.1007/s11657-019-0589-y (PMC6491825; doi:10.1007/s11657-019-0589-y)
Supplement: Supplementary file 1 — (PDF 93 kb) [file 11657_2019_589_MOESM1_ESM.pdf]

Title: Cost-Benefit Analysis of Calcium and Vitamin D Supplements

Submitted to *Osteoporosis International*

Authors: Connie M. Weaver, PhD; Heike A. Bischoff–Ferrari, DrPH; Christopher J. Shanahan

Address for correspondence

Connie M. Weaver, PhD
Distinguished Professor
Department of Nutrition Science
College of Health and Human Sciences
Purdue University
700 W State Street
West Lafayette, IN 47907-2059
Phone: 765-494-8231
Fax: 765-496-9606
E-mail: weavercm@purdue.edu

Online Resource 1. Net cost/savings ratio with combined calcium and vitamin D supplements by (a) EU country (€) and (b) US state (US $)

(a)

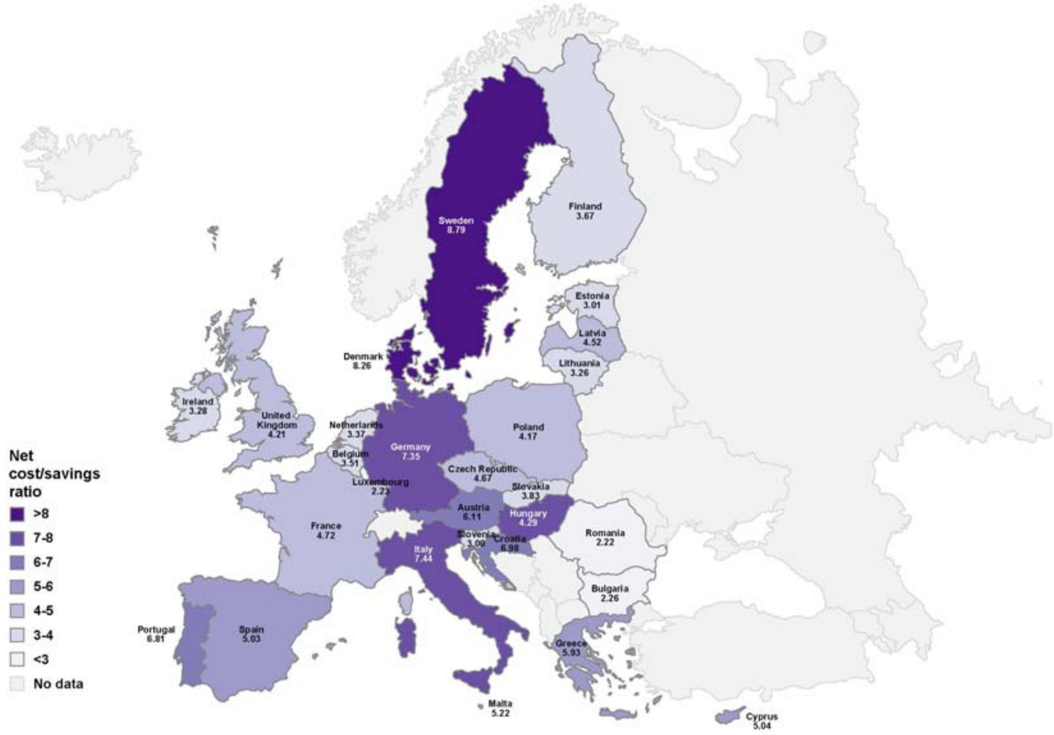

(b)

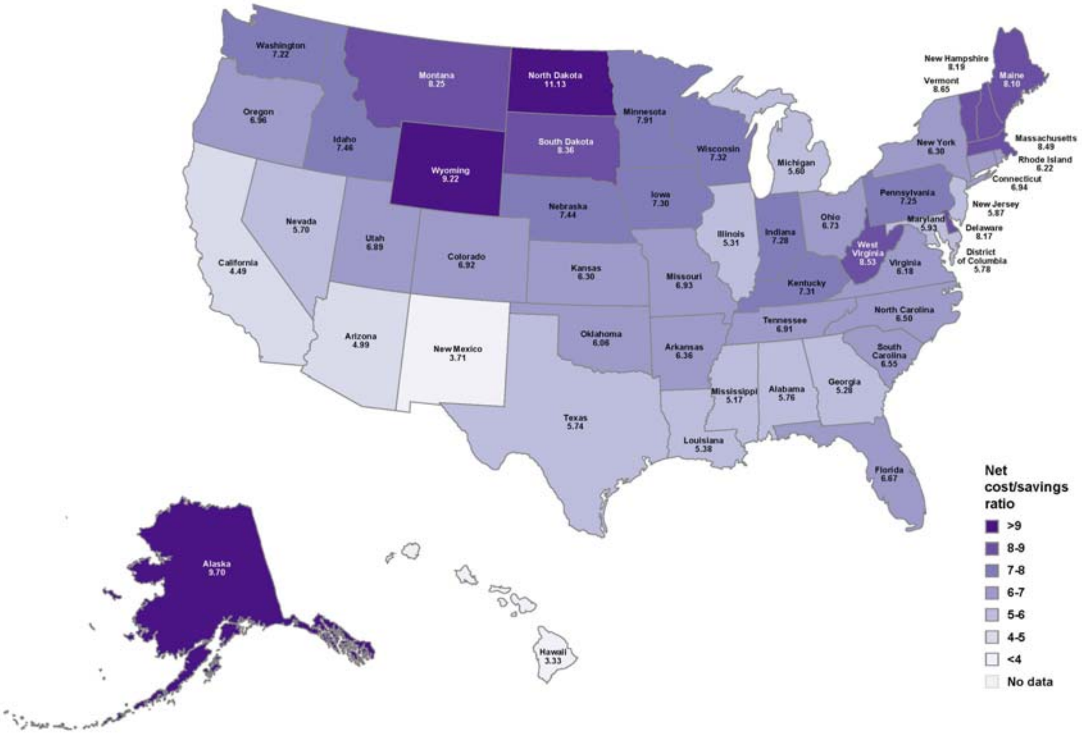
Washington
7.22
Montana
8.25
North Dakota
11.13
Oregon
6.96
Idaho
7.46
Wyoming
9.22
South Dakota
8.36
Minnesota
7.91
Wisconsin
7.32
Michigan
5.60
Iowa
7.30
Nebraska
7.44
California
4.49
Nevada
5.70
Utah
6.89
Colorado
6.92
Kansas
6.30
Missouri
6.93
Illinois
5.31
Indiana
7.28
Ohio
6.73
Kentucky
7.31
West Virginia
8.53
Virginia
6.18
Arizona
4.99
New Mexico
3.71
Oklahoma
6.06
Arkansas
6.36
Tennessee
6.91
North Carolina
6.50
South Carolina
6.55
Texas
5.74
Mississippi
5.17
Alabama
5.76
Georgia
5.28
Louisiana
5.38
Florida
6.67
New Hampshire
8.19
Vermont
8.65
Maine
8.10
New York
6.30
Massachusetts
8.49
Rhode Island
6.22
Connecticut
6.94
Pennsylvania
7.25
New Jersey
5.87
Maryland
5.93
Delaware
8.17
District of Columbia
5.78
Alaska
9.70
Hawaii
3.33
Net cost/savings ratio
>9
8-9
7-8
6-7
5-6
4-5
<4
No data
