# Supplementary material for: Cost-benefit analysis of calcium and vitamin D supplements
Source: Arch Osteoporos. 2019 Apr 30;14(1):50. doi: 10.1007/s11657-019-0589-y (PMC6491825; doi:10.1007/s11657-019-0589-y)
Supplement: Supplementary file 2 — (PDF 17 kb) [file 11657_2019_589_MOESM2_ESM.pdf]

Title: Cost-Benefit Analysis of Calcium and Vitamin D Supplements

Submitted to *Osteoporosis International*

Authors: Connie M. Weaver, PhD; Heike A. Bischoff–Ferrari, DrPH; Christopher J. Shanahan

Address for correspondence

Connie M. Weaver, PhD
Distinguished Professor
Department of Nutrition Science
College of Health and Human Sciences
Purdue University
700 W State Street
West Lafayette, IN 47907-2059
Phone: 765-494-8231
Fax: 765-496-9606
E-mail: weavercm@purdue.edu

Online Resource 2. Calcium and vitamin D supplementation: cost analysis by European Union country, all genders and all age cohorts

Country	*Overall population > 50 years of age, n*	*Number (%) with osteoporosis*	*Number osteoporotic fractures annually*	*Annual fracture incidence, %*	*Total annual cost, €*	*NNT*	*Absolute risk reduction, %*	*Avoided fractures per year, n*	*Avoided hospital costs, €*	*Total annual supplement cost, €*	*Annual net cost benefit, €*
**Austria**	3,567,861	529,881 (14.9)	98,444	18.6	1,049,400,787	39	2.6	13,663	145,642,006	23,840,485	121,801,521
**Belgium**	4,431,423	664,715 (15.0)	87,662	13.2	770,809,507	55	1.8	12,166	106,977,472	30,511,144	76,466,328
**Bulgaria**	2,776,952	402,297 (14.5)	38,769	9.6	119,390,434	75	1.3	5,381	16,569,706	7,346,954	9,222,751
**Croatia**	1,756,869	199,355 (11.3)	38,273	19.2	253,156,509	38	2.7	5,312	35,134,547	5,032,430	30,102,117
**Cyprus**	370,387	49,003 (13.2)	6,160	12.6	66,945,464	57	1.7	855	9,291,085	1,844,377	7,446,707
**Czech Republic**	4,055,023	561,713 (13.9)	80,774	14.4	506,785,063	50	2.0	11,210	70,334,608	15,061,491	55,273,118
**Denmark**	2,199,247	310,714 (14.1)	74,411	23.9	1,075,084,528	30	3.3	10,327	149,206,547	18,074,727	131,131,819
**Estonia**	499,391	78,915 (15.8)	9,301	11.8	55,222,833	61	1.6	1,291	7,664,149	2,546,364	5,117,785
**Finland**	2,268,918	328,767 (14.5)	41,482	12.6	446,430,205	57	1.8	5757	61,958,206	16,883,732	45,074,474
**France**	25,853,783	3,943,485 (15.3)	422,648	10.7	6,094,619,351	67	1.5	58,658	845,847,077	179,217,986	666,629,091
**Germany**	36,569,001	5,507,613 (15.1)	805,959	14.6	12,463,694,895	49	2.0	111,856	1,729,784,797	235,284,186	1,494,500,610
**Greece**	4,458,950	675,123 (15.1)	94,004	13.9	1,022,099,606	52	1.9	13,046	141,852,988	23,932,001	117,920,986
**Hungary**	3,783,878	559,460 (14.8)	107,800	19.3	408,538,855	37	2.7	14,961	56,699,422	13,221,288	43,478,134

Country	*Overall population > 50 years of age, n*	*Number (%) with osteoporosis*	*Number osteoporotic fractures annually*	*Annual fracture incidence, %*	*Total annual cost, €*	*NNT*	*Absolute risk reduction, %*	*Avoided fractures per year, n*	*Avoided hospital costs, €*	*Total annual supplement cost, €*	*Annual net cost benefit, €*
**Ireland**	1,490,821	198,398 (13.3)	21,882	11.0	231,952,040	65	1.5	3037	32,191,667	9,827,978	22,363,689
**Italy**	26,456,172	4,188,589 (15.8)	518,352	12.4	9,587,445,182	58	1.7	71,940	1,330,601,964	178,935,730	1,151,666,234
**Latvia**	795,679	126,549 (15.9)	15,073	11.9	75,280,517	60	1.7	2,092	10,447,872	2,311,101	8,136,772
**Lithuania**	1,164,712	180,404 (15.5)	16,035	8.9	107,029,351	81	1.2	2,225	14,854,162	4,554,030	10,300,132
**Luxembourg**	203,098	28,003 (13.8)	3,234	11.6	22,925,523	62	1.6	449	3,181,739	1,425,358	1,756,381
**Malta**	165,590	21,848 (13.2)	1,736	7.9	27,525,430	91	1.1	241	3,820,141	732,049	3,088,092
**Netherlands**	6,743,456	935,777 (13.9)	88,223	9.4	1,052,430,311	76	1.3	12.244	146,062,461	43,378,425	102,684,037
**Poland**	14,278,120	1,969,543 (13.8)	184,353	9.4	1,372,056,112	77	1.3	25,586	190,422,008	45,649,607	144,772,400
**Portugal**	4,153,807	633,575 (15.3)	58,715	9.3	1,031,168,192	78	1.3	8,149	143,111,579	21,019,489	122,092,090
**Romania**	7,475,626	1,059,862 (14.2)	100,629	9.5	358,252,638	76	1.3	13,966	49,720,406	22,375,816	27,344,590
**Slovakia**	1,933,796	258,400 (13.4)	43,130	16.7	204,054,886	43	2.3	5,986	28,319,936	7,398,335	20,921,601
**Slovenia**	828,271	119,807 (14.5)	18,044	15.1	88,368,418	48	2.1	2,504	12,264,288	4,083,630	8,180,658
**Spain**	18,440,337	2,823,963 (15.3)	236,851	8.4	3,903,614,845	86	1.2	32,872	541,766,600	107,805,201	433,961,400
**Sweden**	3,851,321	575,062 (14.9)	118,336	20.6	1,805,093,870	35	2.9	16,423	250,521,531	28,486,751	222,034,780

Country	*Overall population > 50 years of age, n*	*Number (%) with osteoporosis*	*Number osteoporotic fractures annually*	*Annual fracture incidence, %*	*Total annual cost, €*	*NNT*	*Absolute risk reduction, %*	*Avoided fractures per year, n*	*Avoided hospital costs, €*	*Total annual supplement cost, €*	*Annual net cost benefit, €*
**United Kingdom**	24,180,973	3,554,488 (14.7)	594,380	16.7	5,924,750,837	43	2.3	82,492	822,271,727	195,462,697	626,809,030
**Total EU**	204,753,462	30,485,309 (14.9)	3,924,658	12.9	50,124,126,192	56	1.8	544,687	6,956,520,691	1,246,243,361	5,710,277,330

NNT, number needed to treat
