# Supplementary material for: Cost-benefit analysis of calcium and vitamin D supplements
Source: Arch Osteoporos. 2019 Apr 30;14(1):50. doi: 10.1007/s11657-019-0589-y (PMC6491825; doi:10.1007/s11657-019-0589-y)
Supplement: Supplementary file 3 — (PDF 19 kb) [file 11657_2019_589_MOESM3_ESM.pdf]

Title: Cost-Benefit Analysis of Calcium and Vitamin D Supplements

Submitted to *Osteoporosis International*

Authors: Connie M. Weaver, PhD; Heike A. Bischoff–Ferrari, DrPH; Christopher J. Shanahan

Address for correspondence

Connie M. Weaver, PhD
Distinguished Professor
Department of Nutrition Science
College of Health and Human Sciences
Purdue University
700 W State Street
West Lafayette, IN 47907-2059
Phone: 765-494-8231
Fax: 765-496-9606
E-mail: weavercm@purdue.edu

Online Resource 3. Calcium and vitamin D supplementation: cost analysis by US state, all genders and all age cohorts

US State	Overall population > 50 years of age, n	Number (%) with osteoporosis	Number osteoporotic fractures annually	Annual fracture incidence, %	Total annual cost, US $	NNT	Absolute risk reduction, %	Avoided fractures per year, n	Avoided hospital costs, US $	Total annual supplement cost, US $	Annual net cost benefit, US $
**Alabama**	1,676,350	172,581 (10.3)	37,122	21.5	367,217,201	33	3.0	5,152	50,964,560	8,854,809	42,109,752
**Alaska**	216,224	20,854 (9.6)	5,026	24.1	90,000,773	30	3.3	698	12,490,836	1,288,142	11,202,694
**Arizona**	2,250,923	229,096 (10.2)	48,902	21.3	463,649,979	34	3.0	6,787	64,348,068	12,906,937	51,441,131
**Arkansas**	1,033,441	105,258 (10.2)	24,523	23.3	246,531,059	31	3.2	3,403	34,215,029	5,382,158	28,832,870
**California**	11,660,385	1,192,271 (10.2)	196,991	16.5	2,534,248,608	44	2.3	27,340	351,717,910	78,314,367	273,403,542
**Colorado**	1,757,681	178,315 (10.1)	50,819	28.5	529,835,707	25	4.0	7,053	73,533,712	10,629,085	62,904,627
**Connecticut**	1,258,781	129,550 (10.3)	25,497	19.7	411,828,844	37	2.7	3,539	57,156,026	8,237,322	48,918,704
**Delaware**	332,936	34,817 (10.5)	7,696	22.1	122,016,121	33	3.1	1,068	16,934,114	2,073,257	14,860,858
**District of Columbia**	170,000	18,000 (10.6)	2,412	13.4	51,723,028	54	1.9	335	7,178,426	1,242,376	5,936,049
**Florida**	7,800,893	801,793 (10.3)	181,987	22.7	2,231,941,575	32	3.2	25,257	309,761,964	46,434,222	263,327,741
**Georgia**	3,096,492	319,710 (10.3)	69,824	21.8	653,796,936	33	3.0	9,691	90,737,780	17,188,456	73,549,323
**Hawaii**	511,589	52,359 (10.2)	6,703	12.8	85,777,754	56	1.8	930	11,904,741	3,573,753	8,330,988
**Idaho**	536,188	53,731 (10.0)	15,996	29.8	157,631,057	24	4.1	2,220	21,876,964	2,932,686	18,944,278

US State	Overall population > 50 years of age, n	Number (%) with osteoporosis	Number osteoporotic fractures annually	Annual fracture incidence, %	Total annual cost, US $	NNT	Absolute risk reduction, %	Avoided fractures per year, n	Avoided hospital costs, US $	Total annual supplement cost, US $	Annual net cost benefit, US $
**Illinois**	4,023,241	414,054 (10.3)	72,772	17.6	932,069,104	41	2.4	10,100	129,358,026	24,367,890	104,990,136
**Indiana**	2,173,047	223,251 (10.3)	53,414	23.9	625,178,499	30	3.3	7,413	86,765,945	11,924,680	74,841,264
**Iowa**	1,088,499	111,135 (10.2)	27,404	24.7	308,374,803	29	3.4	3,803	42,798,067	5,864,843	36,933,224
**Kansas**	956,175	97,505 (10.2)	22,039	22.6	234,547,266	32	3.1	3,059	32,551,847	5,168,414	27,383,434
**Kentucky**	1,503,953	155,003 (10.3)	38,320	24.7	422,887,125	29	3.4	5,318	58,690,759	8,034,774	50,655,985
**Louisiana**	1,456,831	149,471 (10.3)	27,888	18.7	309,179,082	39	2.6	3,870	42,909,689	7,983,811	34,925,878
**Maine**	516,623	52,265 (10.1)	12,376	23.7	172,987,831	30	3.3	1,718	24,008,267	2,965,714	21,042,554
**Maryland**	1,958,725	202,883 (10.4)	38,876	19.2	558,413,929	38	2.7	5,395	77,499,965	13,077,973	64,421,992
**Massachusetts**	2,319,994	240,108 (10.3)	54,048	22.5	919,647,356	32	3.1	7,501	127,634,063	15,028,308	112,605,754
**Michigan**	3,325,460	340,518 (10.2)	65,221	19.2	755,854,453	38	2.7	9,052	104,901,921	18,725,749	86,176,173
**Minnesota**	1,821,658	184,929 (10.2)	46,346	25.1	601,420,373	29	3.5	6,432	83,468,652	10,547,783	72,920,870
**Mississippi**	953,321	97,776 (10.3)	17,903	18.3	184,617,273	39	2.5	2,485	25,622,270	4,954,100	20,668,170
**Missouri**	2,089,150	214,539 (10.3)	49,924	23.3	559,492,574	31	3.2	6,929	77,649,666	11,208,339	66,441,327
**Montana**	391,941	39,731 (10.1)	10,976	27.6	129,962,827	26	3.8	1,523	18,037,005	2,187,151	15,849,854
**Nebraska**	624,218	63,480 (10.2)	15,491	24.4	180,097,239	30	3.4	2,150	24,994,953	3,360,899	21,634,054

US State	*Overall population > 50 years of age, n*	*Number (%) with osteoporosis*	*Number osteoporotic fractures annually*	*Annual fracture incidence, %*	*Total annual cost, US $*	*NNT*	*Absolute risk reductio n, %*	*Avoided fractures per year, n*	*Avoided hospital costs, US $*	*Total annual supplement cost, US $*	*Annual net cost benefit, US $*
**Nevada**	941,301	93,901 (10.0)	21,745	23.2	220,249,164	31	3.2	3,018	30,567,473	5,361,580	25,205,893
**New Hampshire**	473,895	48,501 (10.2)	11,608	23.9	176,047,371	30	3.3	1,611	24,432,888	2,981,695	21,451,193
**New Jersey**	2,969,942	307,517 (10.4)	56,442	18.4	870,675,200	39	2.5	7,833	120,837,419	20,576,262	100,261,157
**New Mexico**	722,083	73,595 (10.2)	10,355	14.1	109,079,911	51	2.0	1,437	15,138,751	4,085,964	11,052,787
**New York**	6,477,323	672,040 (10.4)	119,760	17.8	2,062,109,642	40	2.5	16,621	286,191,690	45,440,271	240,751,419
**North Carolina**	3,462,063	358,380 (10.4)	88,030	24.6	899,948,903	29	3.4	12,217	124,900,196	19,205,607	105,694,588
**North Dakota**	259,992	25,999 (10.0)	8,145	31.3	111,485,619	23	4.3	1,130	15,472,629	1,390,240	14,082,389
**Ohio**	4,003,674	411,385 (10.3)	87,037	21.2	1,041,595,318	34	2.9	12,079	144,558,717	21,469,308	123,089,409
**Oklahoma**	1,304,003	133,523 (10.2)	28,771	21.5	307,096,329	33	3.0	3,993	42,620,633	7,030,439	35,590,193
**Oregon**	1,415,200	144,846 (10.2)	34,888	24.1	419,965,569	30	3.3	4,842	58,285,289	8,380,169	49,905,120
**Pennsylvania**	4,671,557	481,783 (10.3)	104,027	21.6	1,444,950,981	33	3.0	14,438	200,538,786	27,649,419	172,889,366
**Rhode Island**	357,127	37,385 (10.5)	6,778	18.1	96,651,955	40	2.5	941	13,413,926	2,156,327	11,257,599
**South Carolina**	1,710,777	176,403 (10.3)	43,347	24.6	440,486,687	29	3.4	6,016	61,133,330	9,329,409	51,803,921
**South Dakota**	296,220	30,271 (10.2)	7,876	26.0	93,803,215	28	3.6	1,093	13,018,561	1,556,688	11,461,874
**Tennessee**	2,296,602	236,334 (10.3)	60,192	25.5	619,875,615	28	3.5	8,354	86,029,979	12,458,404	73,571,575
**Texas**	8,183,184	834,387	186,983	22.4	1,948,723,712	32	3.1	25,951	270,455,325	47,103,542	223,351,783

US State	Overall population > 50 years of age, n	Number (%) with osteoporosis	Number osteoporotic fractures annually	Annual fracture incidence, %	Total annual cost, US $	NNT	Absolute risk reduction, %	Avoided fractures per year, n	Avoided hospital costs, US $	Total annual supplement cost, US $	Annual net cost benefit, US $
		(10.2)									
**Utah**	756,113	76,554 (10.1)	24,735	32.3	215,294,461	22	4.5	3,433	29,879,830	4,339,691	25,540,139
**Vermont**	234,885	24,091 (10.3)	5,713	23.7	88,790,744	30	3.3	793	12,322,901	1,424,820	10,898,081
**Virginia**	2,759,680	283,072 (10.3)	63,900	22.6	756,234,213	32	3.1	8,868	104,954,627	16,972,129	87,982,498
**Washington**	2,440,783	247,698 (10.1)	63,108	25.5	781,711,064	28	3.5	8,758	108,490,454	15,025,375	93,465,079
**West Virginia**	715,213	73,043 (10.2)	17,885	24.5	233,153,875	29	3.4	2,482	32,358,464	3,794,677	28,563,787
**Wisconsin**	1,981,844	200,982 (10.1)	47,148	23.5	578,334,101	31	3.3	6,544	80,264,604	10,969,796	69,294,808
**Wyoming**	210,134	21,237 (10.1)	6,435	30.3	79,347,393	24	4.2	893	11,012,297	1,193,929	9,818,368
**Total US**	106,148,314	10,887,910 (10.3)	2,331,405	21.4	28,436,539,418	34	3.0	323,566	3,946,589,993	634,353,741	3,312,236,252

NNT, number needed to treat; US, United States
