# Supplementary material for: Cost-benefit analysis of calcium and vitamin D supplements
Source: Arch Osteoporos. 2019 Apr 30;14(1):50. doi: 10.1007/s11657-019-0589-y (PMC6491825; doi:10.1007/s11657-019-0589-y)
Supplement: Supplementary file 4 — (PDF 15 kb) [file 11657_2019_589_MOESM4_ESM.pdf]

Online Resource 4. Osteoporosis-attributed fractures and related hospital expenditures in the European Union, by age and gender, 2016–2017

	**Women**				**Men**			
	**50–59 Years of age**	**60–69 Years of age**	**70–79 Years of age**	**≥ 80 Years of age**	**50–59 Years of age**	**60–69 Years of age**	**70–79 Years of age**	**≥ 80 Years of age**
			European Union					
Overall population, n	36,826,222	32,027,703	23,467,622	18,090,522	36,067,839	29,154,940	18,884,394	10,234,220
Number (%) of people with osteoporosis	4,302,219 (11.7)	5,651,342 (17.6)	8,041,580 (34.3)	6,293,926 (34.8)	1,098,766 (3.0)	1,437,737 (4.9)	2,054,448 (10.9)	1,605,291 (15.7)
Number of osteoporosis-attributable fractures/year	457,957	596,067	864,672	664,078	237,824	312,031	448,333	343,695
Annual percentage of osteoporotic population who experience a fracture	10.6	10.5	10.8	10.6	21.6	21.7	21.8	21.4
Total annual hospital costs of osteoporosis-attributed	5,920,743,537	7,528,297,429	11,227,416,901	8,628,677,081	2,965,485,493	4,069,560,241	5,541,753,129	4,242,192,381

fractures,€[a]								

[a]Per-person hospital costs of fracture multiplied by number of osteoporosis-attributable fractures per year
